# Relative roles of land- and ocean-atmosphere interactions in Asian-Pacific thermal contrast variability at the precessional band

**DOI:** 10.1038/srep28349

**Published:** 2016-07-06

**Authors:** Yue Wang, ZhiMin Jian, Ping Zhao, Dong Xiao, JunMing Chen

**Affiliations:** 1 State Key Laboratory of Marine Geology, Tongji University, Shanghai, 200092, China; 2 State Key Laboratory of Severe Weather, Chinese Academy of Meteorological Sciences, Beijing 100081, China; 3 Collaborative Innovation Center on Forecast and Evaluation of Meteorological Disasters, Nanjing University of Information Science and Technology, Nanjing, 210044, China

## Abstract

In a 250-kyr transient simulation of the Community Earth System Model (CESM), we identified a precessional forced seesaw of the summer middle-upper tropospheric eddy temperature between Asia and the North Pacific as the paleo-APO (Asian-Pacific oscillation). The paleo-APO variability is out of phase with the precession parameter. Corresponding to a positive paleo-APO phase, both the subtropical anticyclonic circulation over the North Pacific and the East Asian summer monsoon (EASM) strengthen. Summer anomalous sea surface temperature shows a western cold-eastern warm pattern over the extratropical North Pacific and a zonal positive-negative-positive pattern over the tropical Pacific. The variations in the simulated paleo-APO and East Asian southerly wind at the precessional band agree well with the geological proxies at the Dongge, Sanbao, Linzhu, and Hulu caves in China, which also implies that these proxies may well reflect the variability in the southerly wind over East Asia. Sensitivity experiments further reveal that the reduced precession parameter may enhance the positive paleo-APO phase and the associated EASM because of the response of the land-atmosphere interactions to the precessional insolation changes. The effect of the ocean-atmosphere interactions on the paleo-APO is secondary.

One of the primary sources of the earth’s energy is the solar radiation at the top of the atmosphere, and this radiation is significantly modulated by the earth’s orbital parameters (precession, obliquity and eccentricity). The seasonal distribution of insolation changes is most influenced by the precession parameter (e × sin ω, or climatic precession)^1^,^2^. The increased boreal summer insolation at the precessional band generally strengthens both the land-sea thermal contrasts and the associated summer monsoon circulations in the Northern Hemisphere^3^,^4^,^5^, which suggests a synchronous response of the global monsoon system^6^,^7^,^8^. One response of the East Asian summer monsoon (EASM) circulations may be characterized by intensified surface pressure gradients (or land-sea thermal contrasts) between the East Asian continent and the adjacent oceans and lower-tropospheric southerly wind anomalies over East Asia^9^,^10^. Thus, the lower-tropospheric meridional winds over East Asia may be used to indicate the intensity of the EASM^9^,^11^,^12^, and are highly correlated with the δ^18^O values of cave speleothem records in China and with the precipitation in northern China at the precessional band^11^.

Many studies have examined the regional and seasonal changes in the Asian summer monsoon at orbital timescales^10^,^11^,^13^,^14^,^15^,^16^,^17^,^18^,^19^,^20^. Multiple proxy records indicate the asynchronous evolution of precipitation in different EASM regions during the Holocene^21^,^22^,^23^,^24^,^25^,^26^. Simulation studies have shown that these changes in Asian summer monsoon precipitation are associated with anomalies of the upper-tropospheric westerly wind jets and the subtropical high pressure over the northwestern Pacific^14^,^19^,^27^,^28^, land-air interactions near the Tibetan Plateau^22^,^29^,^30^ and the El Nino-Southern Oscillation (ENSO) events^18^,^31^,^32^,^33^. In particular, the subtropical high pressure is crucial to the intensity and location of the EASM rain belt^20^,^34^, and is forced locally and remotely by changes in diabatic heating over the Asian-Pacific region at the precessional band^27^.

Recent studies have shown that during the modern boreal summer, the atmospheric thermal contrasts between the Asian continent and the North Pacific may be accurately indicated by an extratropical zonal teleconnection index, termed the Asian-Pacific Oscillation (APO)^35^,^36^. Anomalies of APO-like thermal contrasts may have caused decadal-centennial-scale variations in the hydroclimate over Asian monsoon regions during the past millennium^37^,^38^,^39^,^40^,^41^,^42^ and modulated the variability in the subtropical high pressure over the northwestern Pacific and EASM precipitation during the mid-Holocene^43^,^44^. However, the evolution of summer tropospheric thermal contrasts between Asia and the Pacific at the precessional band and their relationships with EASM anomalies have not been investigated. Therefore, we sought to determine whether APO-like land-sea thermal contrasts also appear at the precessional band. If they do, how are the APO-like thermal contrasts at the precessional band (here named the paleo-APO) associated with the EASM? How are they modulated by land- and ocean-atmosphere interactions under the precessional insolation forcing?

With these questions in mind, we investigated the precessional evolution of the tropospheric thermal contrasts between Asia and the Pacific, as well as the associated EASM circulation and precipitation using a transient simulation of the Community Earth System Model (CESM). We also examined the relative roles of land- and ocean-atmosphere interactions in the precessional evolution of the paleo-APO and EASM using the equilibrium simulations of CESM and the Community Atmosphere Model version 4 (CAM4).

## Results

### A transient simulation of the paleo-APO and the associated EASM at the precessional band

**Figure 1:**
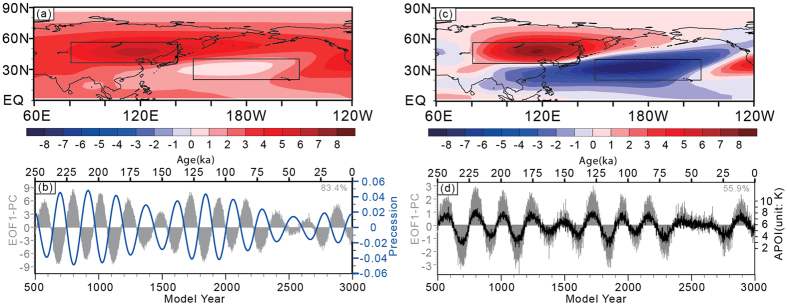
(**a**) The EOF1 mode of JJA 500-200 hPa mean air temperature (*T*) over the Asian-Pacific region in the CESM_transient experiment; (**b**) the time series for Fig. 1a (EOF1-PC, gray bars) and the precession parameter (blue line); (**c**) the EOF1 mode of 500-200 hPa mean eddy temperature (*T′* ); and (**d**) the time series for Fig. 1c (EOF1-PC, gray bars) and the APOI (black line). Figure 1a,c are created using the Grid Analysis and Display System (GrADS) Version 2.0.2, which is available at http://www.iges.org/grads/grads.html. Figure 1b,d are created using the Mircosoft Excel for Mac 2011 (Version 14.5.9, http://www.apple.com/shop/browse/campaigns/office) and combined with Fig. 1a,c in Adobe illustrator CS6 (Version 16.0.0, https://www.adobe.com/downloads.html).

Following on from previous studies^35^,^36^, we performed an empirical orthogonal function (EOF) analysis on summer (June-July-August, hereafter JJA) 500–200 hPa mean temperature (*T*) over the Asian-Pacific region (0°N-90°N, 60°E-120°W), in which temperature data were from the 2500 model years’ output of a CESM transient simulation (denoted experiment CESM_transient, see Methods). The results show that the leading EOF mode (EOF1) (Fig. 1a) accounts for 83.4% of the total variance and exhibits a large-scale warming feature over the study region; however, there is a remarkable difference in the warming magnitude between Northeast Asia (values between 5 and 6) and the northwestern central Pacific (values between 1 and 2). The time series of EOF1 (EOF1-PC) shows significant precessional cycles and is out of phase with the precession parameter (Fig. 1b) with a correlation coefficient of −0.92 (degree of freedom = 22, significant at the 99% confidence level).

To isolate the variation in the Asian-Pacific thermal contrast from the large-scale warming at the precessional band, we further examined the EOF1 of the JJA 500–200 hPa mean eddy temperature (*T′* ), in which *T′* is defined as the difference between *T* and its zonal mean value over the study region. The EOF1 of *T′* (Fig. 1c) accounts for 55.9% of the total variance, with higher values over Northeast Asia between 40°N and 60°N and lower values over the northwestern central Pacific between 20°N and 40°N, which forms a temperature gradient similar to that in Fig. 1a. Moreover, in Fig. 1c, positive anomalies occupy the East Asian continent, and negative anomalies extend from the North Pacific to the southern part of East Asia, which exhibits an APO-like pattern^43^ (here called the paleo-APO). The time series of *T′* EOF1 (Fig. 1d) also shows significant precessional fluctuations and has a correlation of −0.89 (degree of freedom = 22) with the precession parameter. Referring to the EOF1 patterns in Fig. 1a,c, the paleo-APO index (APOI) is defined as the difference in JJA 500–200 hPa *T′* between East Asia (35°N-55°N, 80°E-140°E) and the North Pacific (20°N-40°N, 150°E-150°W). It is evident that this difference is also equal to the difference in JJA 500–200 hPa *T* between these two regions. APOI is highly correlated to the time series of *T′* EOF1 with a correlation coefficient of 0.99 (degree of freedom = 22) and is anti-correlated to the precession parameter with a correlation coefficient of −0.86 (degree of freedom = 22). Thus, when the boreal summer/winter insolation reaches the maximum/minimum at the precessional band (corresponding to the minimum of the precession parameter), the upper-tropospheric land-sea thermal difference indicated by the paleo-APO is enhanced.

**Figure 2:**
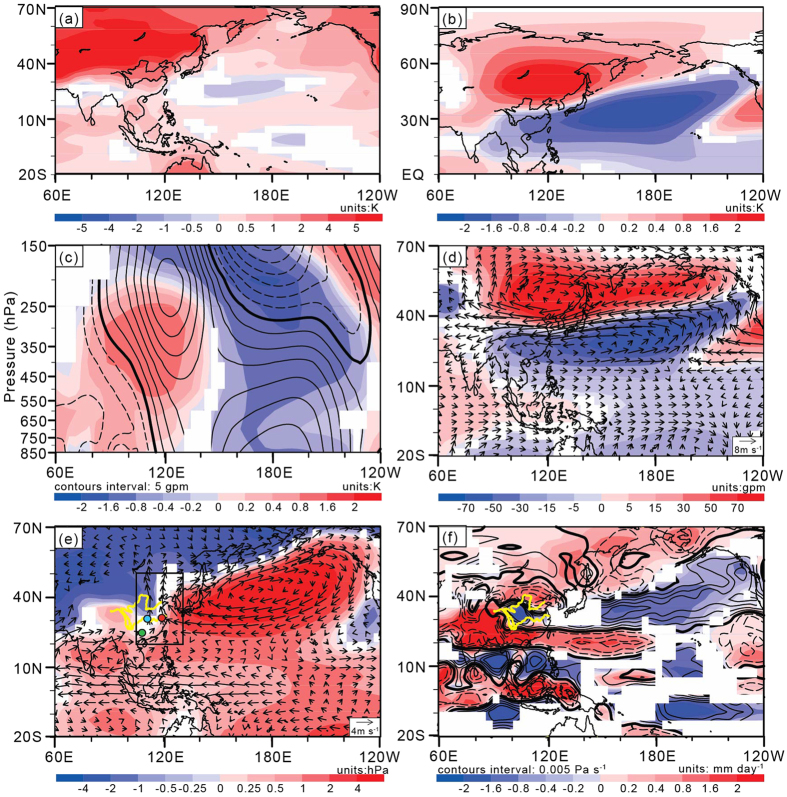
Regression coefficients of JJA atmospheric variables against the normalized APOI: (**a**) surface air temperature (SAT); (**b**) 500–200 hPa mean *T′*; (**c**) longitude-height cross-sections of *T′* (shaded) and eddy geopotential height (*H′*; contour) along the latitudes 20°N-60°N; (**d**) *H′* (shaded) and horizontal winds (vector) at 200 hPa; (**e**) surface pressure (PS; shaded) and horizontal winds (vector) at 850 hPa; and (**f**) precipitation (shaded) and 850–600 hPa mean vertical *p*-velocity (contour). White shaded areas in (**a–f**) are not significant at the 99% level with Student’s *t*-test. In (**c,f**), positive/negative values are plotted using solid/dashed lines, and zero lines are shown as thick solid lines. Black circled dots in (**e**) show the cave locations of stalagmite δ^18^O records, in which the Sanbao and Linzhu caves are blue filled, the Hulu cave is red filled and the Dongge cave is green filled. The Yangtze and Yellow Rivers are plotted as yellow lines. Figure 2a–f are created using GrADS (Version 2.0.2, http://www.iges.org/grads/grads.html) and combined in Adobe illustrator CS6 (Version 16.0.0, https://www.adobe.com/downloads.html).

Figure 2a illustrates the regression coefficient of JJA surface air temperature (SAT) against the normalized APOI at the precessional band. Corresponding to a higher APOI value, positive SAT anomalies between 4 K and 5 K appear over the middle and high latitudes of the Asian continent, and negative SAT anomalies of −0.5 K appear over the Northwest Pacific between 20°N and 40°N. Additionally, positive anomalies (1.6 K to 2 K) of 500–200 hPa mean *T′* appear over the midlatitudes of Asia with negative anomalies between −1.6 K and −2 K over the North Pacific (Fig. 2b). These positive and negative *T′* anomalies are deep in the troposphere, where their central values appear above 500 hPa (Fig. 2c). According to both the vertical motion equation in the static equilibrium case and the equation for air state, an increase in temperature in an air column is associated with an increase in geopotential height at the top of the column and a decrease in geopotential height at the bottom owing to expansion of the air column with locally strengthened upward motion^36^, and vice versa. Thus, the eddy geopotential height (*H′* ) anomalies are a feature of upper-tropospheric positive anomalies and lower-tropospheric negative anomalies between 80°E and 140°E, and the opposite pattern of *H′* appears between 140°E and 140°W (Fig. 2c). At 200 hPa (Fig. 2d), positive/negative *H′* anomalies accompany an anomalous anticyclonic/cyclonic circulation over the midlatitudes of Asia and the North Pacific/lower latitudes of the North Pacific. Moreover, positive anomalies of surface pressure (PS) between 2 hPa and 4 hPa and lower-tropospheric anticyclonic circulation anomalies appear over the North Pacific, with southerly wind anomalies prevailing over the middle and high latitudes of East Asia (Fig. 2e). The southerly wind anomalies strengthen the transport of water vapor, indicating a stronger than normal EASM^45^.

**Figure 3:**
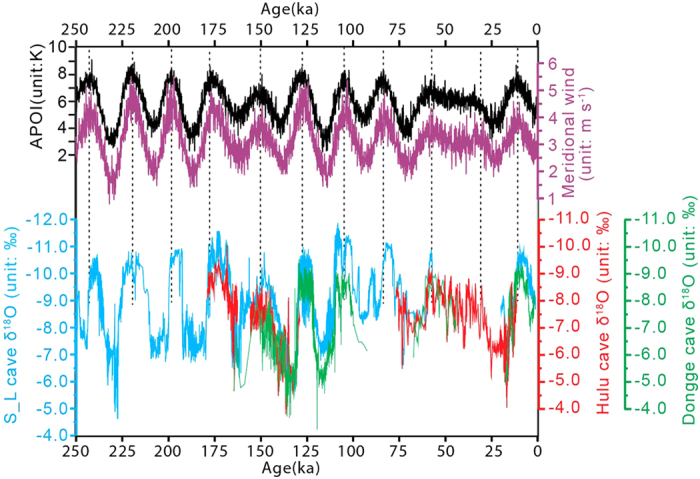
Time series of the simulated APOI (black line) and the regional (20°N-50°N, 105°E-130°E) mean 850-hPa meridional wind (*v*) (purple line) in the CESM_transient experiment and the oxygen isotope (δ^18^O) records of stalagmites in the East Asian caves. Blue lines are for the S-L cave (Sanbao cave at 31°31′N, 110°19′E and Linzhu cave at 31°40′N, 110°26′E)^48^,^51^; red lines are for the Hulu cave at 32°30′N, 119°10′E^47^,^50^; and green lines are for the Dongge cave at 25°17′N, 108°5′E^49^,^52^.

To make a comparison with geological proxies, we calculated the regional (20°N-50°N, 105°E-130°E) mean meridional wind (*v*). The meridional wind is always positive for the entire study period, which indicates a prevailing southerly wind. In Fig. 3, the time series of the southerly wind shows significant precessional cycles and is highly correlated with the APOI with a correlation coefficient of 0.84 (degree of freedom = 22). The Blackman-Tukey method in the Analyseries software^46^ is used to calculate the phase angles of the southerly wind and the APOI against the precession parameter maximum. The southerly wind has a phase angle of −170° (or lags behind the precession parameter minimum by 10°), and the APOI has a phase angle of 178° and leads the southerly wind by 12°. Thus, the APOI may also be taken as an indicator of the EASM intensity at the precessional band. The maximums of both the southerly wind and the APOI are well consistent with the minimums of cave speleothem δ^18^O records in China (shown in Fig. 3)^47^,^48^,^49^,^50^,^51^,^52^. Moreover, *Shi ZG et al.*^18^ suggested that the minimum of speleothem δ^18^O records in China (indicating a stronger EASM) lags behind the precession parameter minimum by 45°, which indicates a phase lag of 35° between the speleothem δ^18^O minimum (phase angle = −135°) and the southerly wind maximum (phase angle = −170°). This relationship between the geological proxies and the simulated southerly wind supports previous conclusions^9^,^11^ and demonstrates the reliability of the simulated atmospheric circulation in this study.

Furthermore, corresponding to a higher APOI value, positive anomalies of the vertical *p*-velocity (corresponding to a descending motion) in the lower troposphere appear between the Yangtze and Yellow Rivers and over the North Pacific between 30°N and 50°N. The negative anomalies (corresponding to ascending motions) appear over the Tibetan Plateau, the midlatitudes of Asia and the tropics from East Asia to the Northwest Pacific (Fig. 2f). Accordingly, there is more rainfall over most parts of East Asia and less rainfall between the Yangtze and Yellow Rivers (Fig. 2f). This rainfall anomaly pattern exhibits a meridional positive-negative-positive mode over the East Asian monsoon region, which indicates a strong EASM^45^,^53^.

### A comparison between land- and ocean-atmosphere interactions

**Figure 4:**
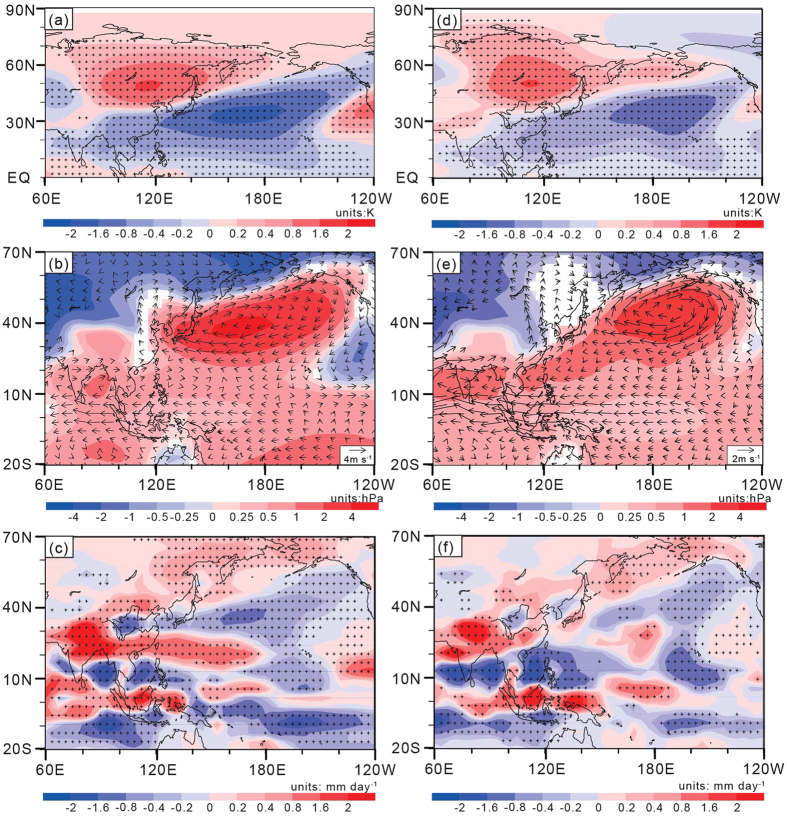
Composite differences in JJA atmospheric variables between the CESM_P_min_ and CESM_control experiments: (**a**) 500–200 hPa mean *T′*; (**b**) surface pressure (PS: shaded) and horizontal winds (vector) at 850 hPa; and (**c**) precipitation (shaded). (**d–f**) are the same as in (**a–c**) but for composite differences between experiments CAM_P_min_ and CAM_control. In (**a**,**c**), (**d**,**f**), the areas marked with crosses are significant at the 99% level with Student’s *t*-test, and in (**b,e**), the white shaded areas are not significant at the 99% level with Student’s *t*-test. Figure 4a–f are also created using GrADS (Version 2.0.2, http://www.iges.org/grads/grads.html) and combined in Adobe illustrator CS6 (Version 16.0.0, https://www.adobe.com/downloads.html).

To examine the effects of the precessional insolation changes on the paleo-APO and the associated EASM anomalies, we conducted two equilibrium experiments using the CESM model with different precessional insolation values (see Methods). Figure 4a–c shows the composite differences in the JJA atmospheric variables between the CESM_P_min_ and CESM_control experiments. In response to the precession parameter minimum, there are significant positive anomalies (0.4 to 0.8 K) of the middle-upper tropospheric *T′* over the midlatitude of Asia and negative anomalies (−0.8 to −1.6 K) over the mid and lower latitudes of the Northwest Pacific (Fig. 4a). These anomalies strengthen the summer land-sea temperature gradient between Asia and the North Pacific. Corresponding to this anomalous pattern of *T′*, positive PS anomalies and lower-tropospheric anticyclonic circulation anomalies appear over the North Pacific (Fig. 4b), with the southerly wind anomalies and positive-negative-positive precipitation anomaly pattern over the EASM region (Fig. 4b,c). These features are similar to those in the CESM_transient experiment (Fig. 2) and highlight a precessional forcing to the paleo-APO and associated EASM in the CESM model.

To separate the influences of land-atmosphere interactions from those of ocean-atmosphere interactions, we additionally conducted two similar equilibrium experiments using CAM4, which is the atmospheric component of CESM (see Methods). Figure 4d–f show the composite differences in JJA atmospheric variables between the CAM_P_min_ and CAM_control experiments. Similar to the CESM model, the precession parameter minimum in CAM4 forces the paleo-APO and the associated atmospheric circulation and rainfall anomalies over the Asian-Pacific region. This result implies that the land-atmosphere coupling system alone can also produce the paleo-APO pattern under precessional forcing. Furthermore, we note that the APOI difference of 1.5 K between the CAM_P_min_ (6.4 K) and CAM_control (4.9 K) experiments is smaller than the difference (1.9 K) between the CESM_P_min_ (7.0 K) and CESM_control (5.1 K) experiments. Does this finding therefore suggest a modulation of ocean-atmosphere interactions for the paleo-APO variability?

**Figure 5:**
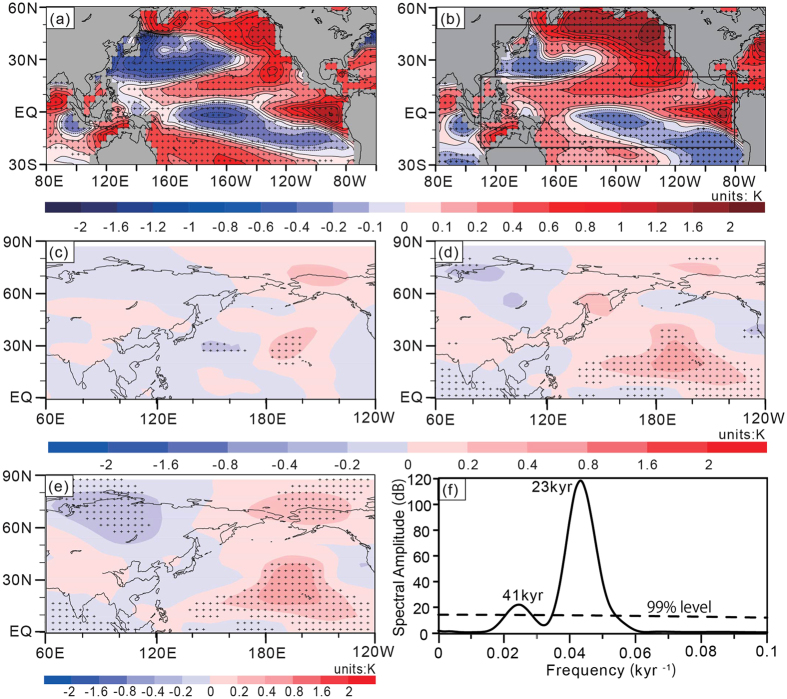
(**a**) Regression coefficients of JJA SST against the normalized APOI in the CESM_transient experiment; (**b**) composite differences in JJA SST between the CESM_P_min_ and CESM_control experiments; (**c**) composite differences of JJA 500–200 hPa mean *T′* between the CAM_P_min__npsst and CAM_P_min_ experiments; and (**d,e**) are the same as in (**c**) but for composite differences between the CAM_P_min__tpsst and CAM_P_min_ experiments and between the CAM_P_min__nptpsst and CAM_P_min_ experiments, respectively; (**f**) spectrum distribution of the APOI in the CESM_transient experiment calculated by Redfit software with a Hanning window, in which dashed lines show the 99% significant level, and the most significant periods are marked by the numbers. Areas marked with crosses in (**a–e**) are significant at the 99% level with Student’s *t*-test. Figure 5a–e are created using GrADS (Version 2.0.2, http://www.iges.org/grads/grads.html) and combined with Fig. 5f (plotted using the Mircosoft Excel for Mac 2011, Version 14.5.9, http://www.apple.com/shop/browse/campaigns/office) in Adobe illustrator CS6 (Version 16.0.0, https://www.adobe.com/downloads.html).

In the modern climate, the APO is positively/negatively correlated with the sea surface temperature (SST) over the extratropical Northwest Pacific/tropical central-eastern Pacific during summer^53^. However, at the precessional band, there is a different relationship between the paleo-APO and the Pacific SST. In Fig. 5a, there is a western cold-eastern warm anomalous pattern with negative SST anomalies over the Northwest Pacific between 20°N and 45°N and positive SST anomalies that are “horseshoe shaped” over the other parts of the North Pacific. This pattern is similar to the winter North Pacific mode (NPM) at the precessional band^54^. Furthermore, a zonal triple anomaly pattern (positive-negative-positive) of JJA SST anomalies dominates the tropical Pacific between 20°S and 20°N (Fig. 5a). The composite difference in JJA SST between the CESM_P_min_ and CESM_control experiments (Fig. 5b) also shows a similar SST anomaly pattern. To examine the effects of these SST anomalies over the Pacific, we also conducted three sensitivity experiments (i.e., CAM_P_min__npsst, CAM_P_min__tpsst and CAM_P_min__nptpsst, see Methods). The results show that the anomalies of JJA 500–200 hPa *T′* forced by the extratropical/tropical Pacific SST anomalies (indicated by the upper/lower black rectangle in Fig. 5b) are generally weaker (0.2 to 0.4 K) (Fig. 5c,d) than those in Fig. 4a,d. The composite difference in 500–200 hPa *T′* between the CAM_P_min__nptpsst and CAM_P_min_ experiments (Fig. 5e), which is a combination of Fig. 5c,d, only results in a weak variation (−0.34 K) of the APOI. Therefore, ocean-atmosphere interactions over the Pacific are not a major contributor to the formation of the paleo-APO at the precessional band.

## Summary and Discussion

Using the CESM model outputs under the transient orbital insolation forcing since 250 ka, we identified a precessional evolution of the summer middle-upper tropospheric thermal contrast between Northeast Asia and the Northwest Pacific, referred to as the paleo-APO. The variability in the paleo-APO is associated with the subtropical high pressure over the North Pacific and the southerly winds over East Asia at the precessional band. The variations in the simulated paleo-APO and East Asian southerly winds are consistent with the geological proxies at the Dongge, S_L and Hulu caves, which suggests that these proxies may indicate the southerly wind variability over East Asia. Corresponding to a stronger than normal paleo-APO, there is less precipitation between the Yangtze and Yellow Rivers and more precipitation over southeastern and northern China. These features indicate an enhanced EASM. The precipitation anomaly pattern is similar to that associated with the recent global warming^45^. Moreover, our results also highlight that the paleo-APO modulates the orbital-scale EASM variabilities^10^ (including southerly winds^9^,^11^,^12^ and precipitation^21^,^22^,^23^,^24^,^25^,^26^ over East Asia).

Summer rainfall is also used as an important climatic factor behind the stalagmite δ^18^O reconstructions (with more rainfall corresponding to more negative δ^18^O)^6^. Here, we further compared the model rainfall with several cave-proxies (such as the Dongge, S_L, and Hulu caves) in the EASM region. The maximums of the simulated JJA rainfall are in good agreement with the negative peaks in δ^18^O at the Dongge cave and are out of phase with those at the S_L cave and Hulu caves (Fig. S1). This result implies that at the precessional band, while the integrated water vapor (with lower δ^18^O values) transported by the enhanced southerly winds can be used to explain the negative δ^18^O shifts in the EASM region^6^,^47^, the local precipitation amount may also largely contribute to the negative δ^18^O peaks at the Dongge cave. The relative contributions of precipitation and wind circulation to stalagmites δ^18^O in different EASM regions should be addressed in future work.

Given that the precessional fluctuations dominate the APOI variability, does this mean that both obliquity and eccentricity also affect the paleo-APO and the associated EASM? Here we analyzed the spectrum feature of the APOI using Redfit software^55^. In addition to the strongest 23-kyr period of precession, the APOI also shows a weaker 41-kyr period of obliquity and does not exhibit a significant 100-kyr period of eccentricity (Fig. 5f). Figure S2a,d further show the responses of the 500–200 hPa mean *T′* to changes in precession and obliquity, respectively. Compared with Fig. S2a, variations in *T′* (Fig. S2d) are weaker and are not significant over the North Pacific; however, there is a slight enhancement of the subtropical high pressure (between 0.5 hPa and 1 hPa) over the North Pacific and weaker southerly winds (Fig. S2e), which are generally consistent with previous studies^13^,^56^. Precipitation anomalies associated with changes in obliquity (Fig. S2f) generally account for approximately 1/3 of those associated with changes in precession (Fig. S2c). The variations in the paleo-APO and EASM associated with eccentricity are generally not significant over the Asian-Pacific region (Fig. S2h,i,j). These results imply that obliquity and eccentricity have weaker effects on the paleo-APO and the associated EASM.

Equilibrium experiments with both CESM and CAM4 further show that the response of the land-atmosphere coupling system alone to the precessional insolation forcing dominates the variations in the paleo-APO and the associated EASM circulation and rainfall anomalies, whereas the effect of ocean-atmosphere interactions on the paleo-APO is secondary. Moreover, a positive phase of the precessional forced paleo-APO is closely associated with a zonal positive-negative-positive pattern of JJA SST in the tropical Pacific and a western cold-eastern warm pattern of JJA SST in the extratropical North Pacific. This relationship between the paleo-APO and SST is different from that of the modern climate.

## Methods

In this study, we used the CESM version 1.0.4 with a resolution of 3.75° for both latitude and longitude for the atmosphere and a nominal resolution of 3° for the ocean^57^. The topography and land-sea distributions and greenhouse gas concentrations are the same as those in 1950 AD. After a spin-up simulation of 200 model years with the fixed orbital insolation of 300 kyrs B.P.^58^, the CESM is integrated for another 3000 model years under the transient orbital insolation forcing of the past 300 kyrs (corresponding to almost thirteen precessional cycles)^1^,^2^. During the last 3000 model years (CESM_transient experiment), the orbital parameters are advanced by 100 years at the end of each model year (that is, with an acceleration factor of 100)^4^,^10^. This CESM_transient experiment was applied in a previous study on the Indian Ocean dipole^58^, and its global annual averaged SAT exhibits a linear increasing trend of 4.04 × 10^−4^ K per model year (Fig. S3a). Considering a possible adjustment time of the CESM atmosphere-ocean system, our analysis are based on the last 2500 model years’ monthly outputs after correcting for the “calendar effect”^59^,^60^ and removing the linear trend. A comparison shows that the results from the processed 2500 model years’ data are similar to those from the 3000 model years’ data before the treatments (shown in Fig. S3a–e). This indicates that the data processing methods have little effect on the results.

We also conducted two equilibrium experiments using the CESM. In the first experiment, the control experiment (CESM_control), solar insolation, topography and land-sea distributions, and greenhouse gas concentrations are set to the value in 1950 AD. With a precession parameter of 0.0169, the summer insolation and winter insolation in the CESM_control experiment stand for a precessional minimum and maximum, respectively. The second experiment (CESM_P_min_) is same as the CESM_control experiment but with a precession parameter of −0.0169, in which the summer insolation and winter insolation reach a precessional maximum and minimum respectively. Each of these two experiments is run for 200 model years. The monthly outputs for the last 100 model years are analyzed. The statistical significance of the composite differences is assessed by the *t*-test at the 99% confidence level.

We then conducted five equilibrium experiments, using the Community Atmosphere Model version 4 (CAM4) that is also the atmospheric component of the CESM. Although coupled with the Community Land Model (CLM, using fixed modern vegetation types) of the CESM, this stand-alone CAM4 includes prescribed modern climatological distributions of SST and sea ice, which means that CAM4 has no ocean-atmosphere interactions. The experiments, CAM_control and CAM_P_min_, have the same configuration of the precessional insolation as the CESM_control and CESM_P_min_ experiments, respectively. The CAM_P_min__npsst, CAM_P_min__tpsst and CAM_P_min__nptpsst experiments have the same configuration of precessional insolation as the CAM_P_min_ experiment. Furthermore, the composite differences in the monthly mean SST over the north Pacific (20°N-50°N, 120°E-120°W)/tropical Pacific (20°S-20°N, 110°E-80°W) between the CESM_P_min_ and CESM_control experiments are added to the CAM_P_min__npsst/CAM_P_min__tpsst experiment. The CAM_P_min__nptpsst experiment is a combination of the CAM_P_min__npsst and CAM_P_min__tpsst experiments. For the aforementioned five experiments, CAM4 is run for 100 model years and the model outputs of the last 50 model years are analyzed.

In this study, all temporal curves were plotted using Mircosoft Excel for Mac 2011 (Version 14.5.9), and the other figures were created using the Grid Analysis and Display System (GrADS), version 2.0.2, which is available at http://www.iges.org/grads/grads.html.

## Additional Information

**How to cite this article**: Wang, Y. *et al*. Relative roles of land- and ocean-atmosphere interactions in Asian-Pacific thermal contrast variability at the precessional band. *Sci. Rep.* **6**, 28349; doi: 10.1038/srep28349 (2016).

## Supplementary Material

Supplementary Information
