# Supplementary material for: Relative roles of land- and ocean-atmosphere interactions in Asian-Pacific thermal contrast variability at the precessional band
Source: Sci Rep. 2016 Jul 6;6:28349. doi: 10.1038/srep28349 (PMC4933979; doi:10.1038/srep28349)
Supplement: Supplementary Information [file srep28349-s1.pdf]

Relative roles of land- and ocean-atmosphere interactions in Asian-Pacific thermal contrast variability at the precessional band

By Yue Wang[1, 2]; ZhiMin Jian[1]; Ping Zhao[2, 3*]; Dong Xiao[2]; JunMing Chen[2]

[1]State Key Laboratory of Marine Geology, Tongji University, Shanghai, 200092, China;

[2]State Key Laboratory of Severe Weather, Chinese Academy of Meteorological Sciences, Beijing, 100081, China;

[3]Collaborative Innovation Center on Forecast and Evaluation of Meteorological Disasters, Nanjing University of Information Science and Technology, Nanjing, 210044, China

Corresponding author E-mail *address*: zhaoping@camscma.cn

Supplementary Figure Captions

Figure S1. The time series for the reconstructed stalagmite oxygen isotope ($\delta^{18}O$, unit: ‰) and the simulated JJA precipitation (unit: mm day^{-1}) from the CESM_transient experiment at the S-L (blue), Hulu (red) and Dongge (green) caves.

Figure S2. Regression coefficients of JJA atmospheric variables against the normalized time series of precession (multiplied by -1): (a) 500-200 hPa mean T'; (b) surface pressure (PS; shaded) and horizontal winds at 850 hPa (vector); (c) precipitation (shaded). (d)-(f)/(h)-(j) same as in (a)-(c) but for obliquity/eccentricity. In (b), (e) and (i), white shaded areas are not significant at the 99% level, and in (a), (c), (d), (f), (h) and (j), the areas marked with crosses are significant at the 99% level. Figure S2a-j are created / combined using GrADS (Version 2.0.2, http://www.iges.org/grads/grads.html) / Adobe illustrator CS6 (Version 16.0.0, https://www.adobe.com/downloads.html).

Figure S3. (a) The time series for global annual averaged surface air temperature (SAT, unit: K; black line), its linear trend (black dashed line), the APOI (unit: K; blue line) and the EOF1 time series of JJA 500-200 hPa mean T' (grey line) using the CESM_transient experiment output before correcting for the "calendar effect" and removing the linear trend since 300 ka; (b) EOF1 mode of 500-200 hPa mean T' and its contribution (%) to the total variance; (c) regression coefficients of JJA SAT against the normalized APOI in (a); (d) same as in (c) but for surface pressure (PS; shaded) and horizontal winds at 850 hPa (vector); (e) same as in (c) but for precipitation. In (d), the white shaded areas are not significant at the 99% level; in (c) and (e), the areas marked with crosses are significant at the 99% level. Figure S3b-e are created using GrADS (Version 2.0.2, http://www.iges.org/grads/grads.html). Figure S3a is plotted using the Mircosoft Excel for

Mac 2011 (Version 14.5.9, http://www.apple.com/shop/browse/campaigns/office) and combined with Figure S3b-e in Adobe illustrator CS6 (Version 16.0.0, https://www.adobe.com/downloads.html).

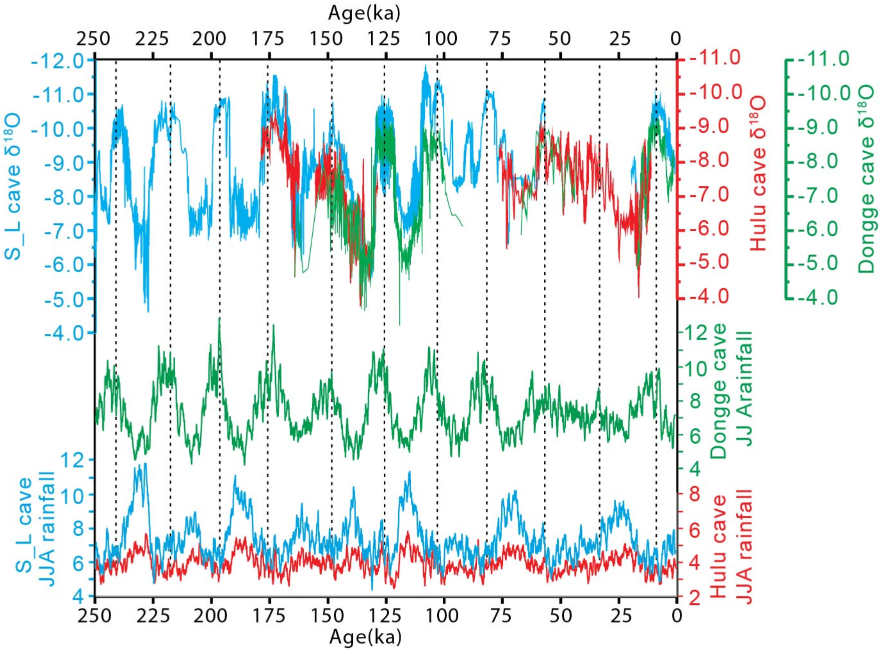

Figure S**1**. The time series for the reconstructed stalagmite oxygen isotope ($\delta^{18}O$, unit: ‰) and the simulated JJA precipitation (unit: mm day^{-1}) from the CESM_transient experiment at the S-L (blue), Hulu (red) and Dongge (green) caves.

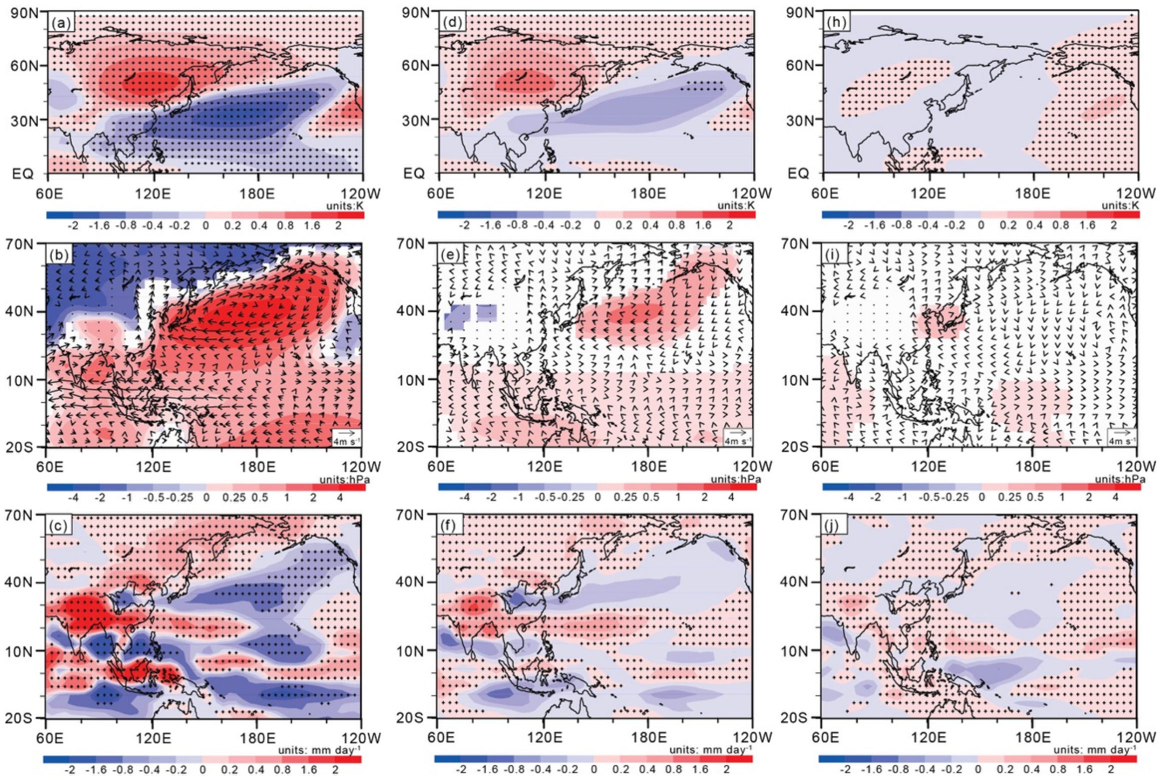

Figure S2. Regression coefficients of JJA atmospheric variables against the normalized time series of precession (multiplied by -1): (a) 500-200 hPa mean *T'*; (b) surface pressure (PS; shaded) and horizontal winds at 850 hPa (vector); (c) precipitation (shaded). (d)-(f)/(h)-(j) same as in (a)-(c) but for obliquity/eccentricity. In (b), (e) and (i), white shaded areas are not significant at the 99% level, and in (a), (c), (d), (f) , (h) and (j), the areas marked with crosses are significant at the 99% level. Figure S2a-j are created / combined using GrADS (Version 2.0.2, http://www.iges.org/grads/grads.html) / Adobe illustrator CS6 (Version 16.0.0, https://www.adobe.com/downloads.html).

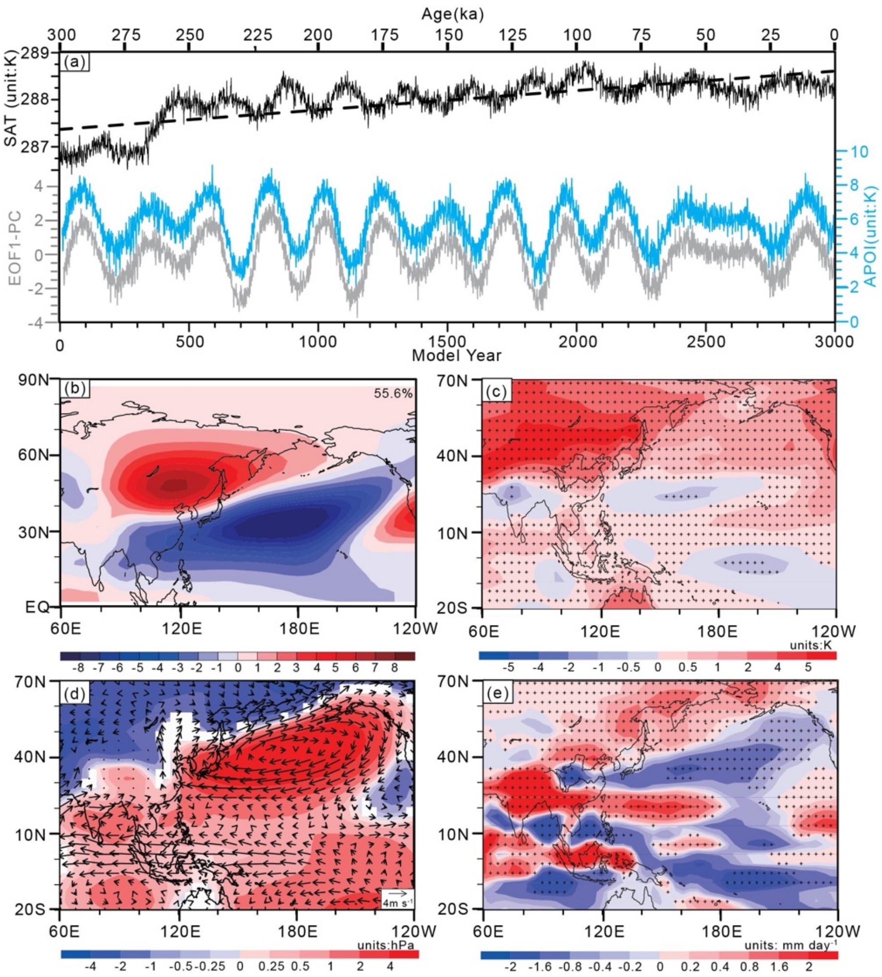

Figure S**3**. (a) The time series for global annual averaged surface air temperature (SAT, unit: K; black line), its linear trend (black dashed line), the APOI (unit: K; blue line) and the EOF1 time series of JJA 500-200 hPa mean *T′* (grey line) using the CESM_transient experiment output before correcting for the "calendar effect" and removing the linear trend since 300 ka; (b) EOF1 mode of 500-200 hPa mean *T′* and its contribution (%) to the total variance; (c) regression coefficients of JJA SAT against the normalized APOI in (a); (d) same as in (c) but for surface pressure (PS; shaded) and horizontal winds at 850 hPa (vector); (e) same as in (c) but for precipitation. In (d), the white shaded areas are not significant at the 99% level; in (c) and (e), the areas marked with crosses are significant at the 99% level. Figure S3b-e are created using GrADS (Version 2.0.2,

http://www.iges.org/grads/grads.html). Figure S3a is plotted using the Mircosoft Excel for Mac 2011 (Version 14.5.9, http://www.apple.com/shop/browse/campaigns/office) and combined with Figure S3b-e in Adobe illustrator CS6 (Version 16.0.0, https://www.adobe.com/downloads.html).
